# Anatomical Dissection in Enlightenment England and Beyond

**Published:** 2014-01

**Authors:** Jenny Skidmore

This book contains a collection of works presenting a view of anatomical dissection and the development of pathology museums through a series of articles by experts in biological anthropology and history. The editor introduces the work of the various authors and leads the reader on a journey following the path taken by the ‘donor’ from their deathbed (or gallows) to their potential resting place as a specimen in a museum collection.

The first part of the book comprises accounts of archaeological excavations of human skeletal remains from workhouses, prisons, private anatomy schools and medical schools across England from the 1600s to the 1800s. It highlights the practices and procedures of anatomical examination and surgical training conducted in Enlightenment England. The extracts from the literature of the time illustrate how anatomical examination was viewed by the public, the profession and those more intimately involved in the procurement of cadavers (the ‘resurrection men’).

Through detailed analysis of catalogues and auction records, the second part of the book explores the development of a number of pathology museums. These were established to educate and display to the public how anatomy changes with disease but they were also showcases for the expertise of the institution and their contents often reflected the interests of various curators. The final article provides an example of the way museum collections may be used to demonstrate how disease was experienced in the past.

As an anatomist, I found this book enlightening and I feel it would be relevant to anyone with an interest in anatomy, surgery or pathology.

